# Face and content validity of the EMPOWER-UP questionnaire: a generic measure of empowerment in relational decision-making and problem-solving

**DOI:** 10.1186/s12911-024-02727-5

**Published:** 2024-10-28

**Authors:** Emilie Haarslev Schröder Marqvorsen, Line Lund, Sigrid Normann Biener, Mette Due-Christensen, Gitte R. Husted, Rikke Jørgensen, Anne Sophie Mathiesen, Mette Linnet Olesen, Morten Aagaard Petersen, François Pouwer, Bodil Rasmussen, Mette Juel Rothmann, Thordis Thomsen, Kirsty Winkley, Vibeke Zoffmann

**Affiliations:** 1 Department of Clinical Medicine, University of Copenhagen, Copenhagen N, 2200 Denmark; 2 Palliative Care Research Unit, Department of Geriatrics and Palliative Medicine GP, Bispebjerg and Frederiksberg Hospital, Copenhagen, NV 2400 Denmark; 3 Department of Nutrition, Exercise and Sports, University of Copenhagen, Copenhagen N, 2200 Denmark; 4 Department of Prevention, Health Promotion and Society, Steno Diabetes Center Copenhagen, Herlev, 2730 Denmark; 5 Department of Research and Development, Danish College of Pharmacy Practice, Pharmakon, Hillerød, 3400 Denmark; 6 Unit for Psychiatric Research, Aalborg University Hospital, 9000 Aalborg, Denmark; 7 Department of Endocrinology, Center for Cancer and Organ Diseases, Copenhagen University Hospital – Rigshospitalet, Copenhagen Ø, 2100 Denmark; 8 The Interdisciplinary Research Unit of Women’s, Children’s, and Families’ Health, the Juliane Marie Centre, Copenhagen University Hospital, Rigshospitalet, Copenhagen Ø, 2100 Denmark; 9 Department of Psychology, University of Southern Denmark, 5000 Odense C, Denmark; 10 The Centre for Quality and Patient Safety, Institute of Health Transformation – Western Health Partnership, Western Health, St Albans, VIC Australia; 11 Steno Diabetes Center Odense, Odense University Hospital, Odense C, 5000 Denmark; 12 Faculty of Nursing, Midwifery and Palliative Care, King’s College London, London, UK; 13 Department of Public Health, University of Copenhagen, Copenhagen K, 1353 Denmark

**Keywords:** Empowerment, Decision making, Problem solving, User-provider relationships, Questionnaire design, Patient-reported outcome measure

## Abstract

**Background:**

Decision-making and problem-solving processes are powerful activities occurring daily across all healthcare settings. Their empowering potential is seldom fully exploited, and they may even be perceived as disempowering. We developed the EMPOWER-UP questionnaire to enable assessment of healthcare users’ perception of empowerment across health conditions, healthcare settings, and healthcare providers’ professional backgrounds. This article reports the initial development of EMPOWER-UP, including face and content validation.

**Methods:**

Four grounded theories explaining barriers and enablers to empowerment in relational decision-making and problem-solving were reviewed to generate a preliminary item pool, which was subsequently reduced using constant comparison. Preliminary items were evaluated for face and content validity using an expert panel of seven researchers and cognitive interviews in Danish and English with 29 adults diagnosed with diabetes, cancer, or schizophrenia.

**Results:**

A preliminary pool of 139 items was reduced to 46. Independent feedback from expert panel members resulted in further item reduction and modifications supporting content validity and strengthening the potential for generic use. Forty-one preliminary items were evaluated through 29 cognitive interviews, resulting in a 36-item draft questionnaire deemed to have good face and content validity and generic potential.

**Conclusions:**

Face and content validation using an expert panel and cognitive interviews resulted in a 36-item draft questionnaire with a potential for evaluating empowerment in user-provider interactions regardless of health conditions, healthcare settings, and healthcare providers’ professional backgrounds.

**Supplementary Information:**

The online version contains supplementary material available at 10.1186/s12911-024-02727-5.

## Plain Language Summary

Users of healthcare services living with long-term health conditions may need empowerment-based support from healthcare providers to discover and develop their inherent capacity to be responsible for their own life. This ensures that healthcare users develop the skills needed to manage the many decisions and problems they face. Yet many healthcare users still experience lack of involvement and support in decision-making and problem-solving. Therefore, we developed the EMPOWER-UP questionnaire to allow healthcare users to evaluate the empowering qualities of their interactions with healthcare providers regardless of their diagnosis, where the interactions take place, and with whom. The aim of this article was to describe the development of EMPOWER-UP and to present the results of early evaluations of its content.

The questionnaire was developed in Danish based on thorough qualitative research explaining distinct, complex patterns in user-provider interactions and translated to English during the evaluation process. We used individual feedback from a panel of expert researchers and interviews conducted with healthcare users diagnosed with diabetes, cancer, or schizophrenia to evaluate EMPOWER-UP’s content. Interviews were conducted while the participants answered preliminary versions of the questionnaire.

As a result of these evaluations, several items in the questionnaire were adjusted, some were deleted, and some new items were developed. We believe that EMPOWER-UP can be an important tool to help evaluate the effectiveness of interventions aimed at ensuring empowering support from healthcare providers. Thus, EMPOWER-UP may help to identify the most effective interventions for specific healthcare contexts.

**Supplementary Information:**

The online version contains supplementary material available at 10.1186/s12911-024-02727-5.

## Introduction

For users of healthcare services living with long-term health conditions (LTHCs), managing the many difficult decisions and problems of daily life often requires support from healthcare providers (HCPs). Empowerment is an important aspect of such support because it may help healthcare users become more autonomous by taking a more active role in decision-making [1, 2]. Empowerment is most often [3] defined as “the discovery and development of [one’s] inherent capacity to be responsible for one’s own life” ([4], p.38]). Arguably, decision-making and problem-solving are among the most powerful activities shared by users and providers of long-term healthcare, with great empowering potential [1, 5–7].

However, healthcare users do not always perceive these activities as empowering. Since empowerment was introduced more than three decades ago as an approach needed in healthcare [4, 8], research has documented the failure to realize empowerment in user-provider interactions, representing a major gap [9–11]. These failures include HCP perceptions of empowerment as something to be given or taken, a tool to be used only in certain cases, or an approach to increase adherence and compliance [2, 5, 10, 12–14]. Consequently, HCPs may inaccurately believe that they already engage in empowerment-based care [13]. This may explain why some healthcare users continue to experience being involved in decision-making in only limited or superficial ways [12, 14]. Although interacting with healthcare users in autonomy-supportive and empowerment-based ways is a high priority, it seems difficult for HCPs to evaluate their abilities to do so [15]. Therefore, the empowering qualities of user-provider interactions related to problem-solving and decision-making should be assessed from the perspective of healthcare users, requiring a user-reported questionnaire.

We consider a questionnaire designed for this purpose should meet four criteria. First, it must focus on barriers to and enablers of empowerment in decision-making and problem-solving processes (criterion 1). As argued by Aujolat, d’Hoore, and Deccache ([1], p.18]), mutual decision-making and problem-solving is needed to acknowledge individual healthcare users’ “experience, priorities, and fears”. Despite HCPs’ good intentions to support individuals with an LTHC, serious barriers in decision-making and problem-solving have been identified and explained by Zoffmann and Kirkevold [6, 16], and Zoffmann, Harder, and Kirkevold [17]. Knowing these barriers, it became possible to overcome them and thereby realise empowerment in clinical practice, first in diabetes [18] and subsequently across multiple LTHCs [19].

A qualitative foundation for questionnaire content is frequently recommended in guidelines on questionnaire development [20–22]. Moreover, it has been suggested that the knowledge base of the construct in question should be theoretically developed in order to develop questionnaires with the potential for construct validity [23]. Such a knowledge base may best be generated through grounded theory research, ideal for illuminating and conceptualising otherwise hidden social processes [24]. Grounded theory has also been suggested as suitable for ensuring content validity [25] and developing a questionnaire applicable as an endpoint in clinical trials [26]. For these reasons, we believe that the proposed questionnaire should build on a conceptual foundation derived from grounded theory methodology (criterion 2). Generic properties in a questionnaire ensure that measurements across various health contexts are comparable [27]. Although some researchers have suggested empowerment as a context-specific construct, most scientific literature seems to lean towards a generic approach [3]. Accordingly, we consider it important that the questionnaire in question should have potential for generic use across LTHCs, HCPs’ professional backgrounds, and healthcare settings including hospital wards, outpatient clinics, general practices, nursing homes, community settings, and healthcare users’ homes (criterion 3).

Finally, we agree with researchers such as Mora et al. [3] and Pekonen et al. [27] who argued that any future questionnaire on empowerment should be meticulously developed and validated to ensure good psychometric properties (criterion 4).

We conducted a systematic literature search in 2018 (updated in 2023) in PubMed to identify questionnaires meeting the four criteria stated above. We searched for questionnaires assessing empowerment or related concepts (i.e., patient engagement, patient enablement, patient activation, and shared decision-making) in user-provider interactions. In addition, we searched for questionnaires assessing the quality of user-provider interactions. No questionnaires met all four criteria, and we designed the EMPOWER-UP questionnaire to do so.

Recent recommendations for best practice in questionnaire development [21] and consensus-based standards for health measurement instruments (COSMIN) [28, 29] suggest that content validity, including face validity, should be evaluated as the first step after generating the questionnaire content. Accordingly, we report here on the initial development of EMPOWER-UP to align with the first three criteria, including face and content validation by representatives of the target population. Psychometric properties of the final questionnaire will be reported separately.

## Methods

Development and validation of EMPOWER-UP in November 2018 – September 2023 followed recommendations by Boateng et al. [21]. It was embedded in pragmatic research, as suggested by Glasgow [30] who builds on Dewey’s iterative-circular understanding of problem-solving, suggesting that measurements ought to be pragmatic to ensure their real applicability [30, 31].

### The conceptual foundation

**Fig. 1:**
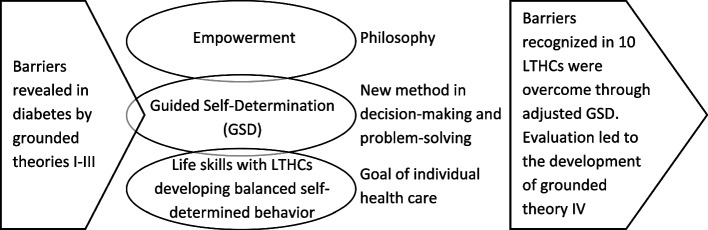
The theoretical framework used in the development of EMPOWER-UP originating from a Danish research program on the Guided Self-Determination method (GSD). Note: Grounded theory I-III had been directive for the development of GSD in diabetes [6, 16, 17] and grounded theory IV was developed through evaluation of GSD in ten long-term health conditions (LTHCs) [33]. GSD functions as a link between the philosophy of empowerment and the goal of life skills

The conceptual framework used for questionnaire development (Fig. 1) was originally used in a Danish intervention program aimed at developing a supplementary decision-making and problem-solving method to address the criticism that traditional methods were inconsistent with empowerment [32]. Researchers studied user-provider interactions during decision-making and problem-solving activities between healthcare users with diabetes and with persistent glucose levels above recommended targets and their HCPs, illuminating barriers and enablers to related empowerment (meeting criterion 1). To understand the mechanisms behind these findings, they developed grounded theories (Fig. 1, grounded theory I-III) explaining barriers and enablers in usual care (meeting criterion 2) [6, 16, 17], which directed the development of the supplementary Guided Self-Determination decision-making and problem-solving method. Although the framework was developed in the context of diabetes, it was assumed to have a strong potential for generic application (meeting criterion 3), as indicated by a pragmatic [31] grounded theory (Fig. 1, grounded theory IV) explaining similar findings across 10 various LTHCs [33]. The success of the broad application is likely due to the deliberate choice to develop Guided Self-Determination in the context of the most challenging interactions between users with type 1 diabetes and HCPs [32]. This context was selected to optimize the capacity of Guided Self-Determination to overcome barriers in challenging interactions between users with other LTHCs and HCPs.

The framework applied Anderson and Funnell’s definition of empowerment, in which empowerment is the healthcare users’ ability to discover and develop their “inherent capacity to be responsible for their own life” ([3], p.38]). Empowerment constitutes the *philosophy* of the framework and Guided Self-Determination is the *method,* whereas developing life skills was the *goal*, aligned with the Ottawa Charter’s identification of life skills [34, 35] as a meaningful goal for individuals in long-term care. As defined by Mullen [36], life skills are “problem solving behaviours used in the management of personal affairs. They apply to five areas of life responsibility: Self, Family, Leisure, Community, Job” (p. 84).

Table 1 summarizes mechanisms important to empowerment during decision-making and problem-solving explained by the foci of the four grounded theories: GT I, a life-versus-disease conflict [6]; GT II, characteristics of the user-provider relationship [16]; GT III, the quality of communication and reflection during decision-making [17]; and GT IV, the empowering potential of person-specific evidence [33]. In addition, Table 1 depicts the generic applicability of the theoretical framework used to develop EMPOWER-UP documented through the wide dissemination of Guided Self-Determination, which was developed from the same foundation.

**Table 1:** Relevance and potential generic use of grounded theories I-IV for understanding and intervening in (dis)empowerment across long-term health conditions

Grounded theories (GTs) explained barriers and enablers in diabetes care for healthcare users with persistent glucose levels above recommended targets			Intervention studies across > 10 LTHCs	Pragmatic grounded theory explained the power of person-specific evidence across > 10 LTHCs in GSD interventions
Participants across the three GTs: 11 healthcare user-nurse dyads **66 qualitative data sources:** Observations of admission interviews, collegial discussions, and discharge interviews Semi-structured interviews with healthcare users and subsequently with nurses immediately after discharge Semi-structured interviews with healthcare users again six months after discharge			Patterns revealed by GT I-III directed the development of Guided Self-Determination (GSD) as a supplementary decision-making and problem-solving method through participatory action research [32] involving 25 healthcare users and 12 nurses. GSD was qualitatively demonstrated to change the patterns in an intervention study [18] matching participants and data sources from GT I-III The same patterns were recognised by researchers and clinicians in other areas of long-term health conditions (LTHCs), thereby demonstrating GSD’s ability to change the patterns across LTHCs **Impact of GSD tested in:** 12 RCTs, 26 qualitative studies, 3 mixed methods studies, 1 non-randomized feasibility study, and 1 inspired by participatory implementation, identified in an integrative review [19] Additionally, 2 qualitative studies [37, 38], 1 sequential two-phase multiple method feasibility study [39] **LTHCs:** Diabetes, neonatal care, schizophrenia, intensive care survivors, gynaecologic cancer, breast cancer, acute stroke, chronic pain, ADHD, eating disorders, end-stage kidney disease, endometriosis **Settings:** Hospital wards, outpatient clinics, assertive outreach teams, intensive care units, municipal rehabilitation units, dialysis units, pain centres, in-home care, general practices, primary care, online platform, community care	GT-IV [33] was developed as a pragmatic GT comparing examples from 20 intervention studies using GSD as a supplementary method across 10 various LTHCs. The theory explains the power of person-specific evidence created through empowering insight rather than traditional narrow disease-specific knowledge or unverified assumptions about each person in decision-making and problem-solving Moreover, the theory showed the ability of person-specific evidence to mobilize relational capacities in everyone involved in an individual healthcare user’s situation **LTHCs:** Diabetes, diabetes and comorbid eating disorder, neonatal care, schizophrenia, gynaecologic cancer, breast cancer, infertility, ADHD, COPD, end-stage kidney disease, endometriosis, multiple psychiatric conditions **Settings:** Hospital wards, outpatient clinics, intensive care units, assertive outreach teams, municipal rehabilitation units, dialysis units, general practices
GT-I [6] explains patterns related to a life-versus-disease conflict between healthcare users and their healthcare provider. In traditional interactions, this conflict remains mostly unchanged—or even deadlocked—instead of being resolved	GT-II [16] distinguishes between three kinds of relationships between healthcare users and their healthcare provider It explains why a relationship characterized by mutuality makes room for releasing a potential for change, in contrast to typical relationship types characterized by provider dominance or blurred sympathy	GT-III [17] developed a model distinguishing between one focused and four de-focused communication zones, as well as four depths of reflection. The theory explains the necessity of reaching focused communication and deep mutual reflection to establish mutual understanding in the shared decision-making and problem-solving process		

*Abbreviations: ADHD* attention deficit hyperactivity disorder, *COPD* chronic obstructive pulmonary disorder, *GT* grounded theory, *LTHC* long-term health condition, *RCT* randomized controlled trial

The comprehensiveness of GT I-III is supported by the use of multiple data sources including observations and repeated interviews with healthcare users, as well as HCPs following dyads over more than six months (Table 1). The extensive investigation of the perspectives of both healthcare users and HCPs in the grounded theory analyses is indispensable for developing a valid and reliable questionnaire applicable across clinical settings (meeting criterion 4).

### Domain identification and item generation

The overall domain of EMPOWER-UP was identified as *empowerment in relational decision-making and problem-*solving. As the conceptual framework chosen as the foundation for the questionnaire was originally developed in Danish, we decided first to develop the questionnaire in Danish and subsequently in English. To generate a preliminary item pool in Danish, we conducted a conceptual review of data from GT I-III reports [6, 16, 17], a theory-driven qualitative evaluation of the Guided Self-Determination method used in Danish diabetes care [18], and the development of GT IV [32, 33]. Additionally, we consulted a code book originally generated by VZ during GT I-III data analysis.

The preliminary item pool was subjected to constant comparison by VZ and EM to choose items that best covered the distinct relational patterns from the theories. We removed duplicates and closely related items because VZ, as the primary investigator on all four grounded theories, could appropriately prioritise them. In addition, early item reduction provided a more manageable number of items for expert panel and cognitive evaluations.

### Expert panel evaluation

We invited seven Danish researchers to participate in an expert panel [21] evaluating content validity of the reduced pool of preliminary items [29]. Six researchers were selected based on their knowledge of the conceptual framework (RJ, ASM, MLO, MD, GRH, JW) and broad clinical experience with its use in intervention studies in various conditions (schizophrenia, benign and malignant gynaecological conditions, type 1 and 2 diabetes, and parenting extremely premature infants). Additionally, LL was selected based on her expertise in questionnaire development. Panel members were asked to provide individual written feedback on item comprehensiveness, relevance, representativeness, and potential for generic use. EM and VZ reviewed the panel feedback and accepted, rejected, or modified suggested changes based on findings and convincing arguments. Finally, they compared the revised item sets with key concepts from the conceptual framework to ensure full construct coverage.

### Cognitive interviews

#### Participants and recruitment

To assess content and face validity [21, 29], we invited healthcare users aged ≥ 18 years diagnosed with diabetes, cancer, or schizophrenia from Denmark, the United Kingdom, and Australia to participate in cognitive interviews [40, 41]. Healthcare users were eligible to participate if they had an experience of interacting with HCPs related to one of these diagnoses within the previous 6–12 months. Individuals with active psychosis as assessed by HCPs were excluded. These LTHCs were selected to include the perspectives of individuals with a range of complex conditions.

Danish-speaking participants were purposely recruited from two patient organisations and inpatient hospital wards and outpatient clinics in the Capital Region and Region Zealand between July 2019 and March 2020. English-speaking participants were recruited in July–August 2020 via clinical research networks at Deakin University (Melbourne, Australia) and King’s College London (United Kingdom). EM or VZ approached key stakeholders from each institution who subsequently invited eligible healthcare users.

#### Interviewing technique

Interviews were initially carried out in Danish in iterative rounds with 1–7 participants until no new insights emerged. Notes about the observed problems with specific items were taken by EM after each interview and discussed with VZ. Each round was ended when EM and VZ agreed that revisions to one or more items were needed, independent of the number of participants interviewed. After the final round, the revised draft questionnaire was cross translated [42] into English, and additional interview rounds were conducted in English to ensure that no new insights emerged. Each interviewing round evaluated a revised draft of the questionnaire.

All participants were asked to complete a draft version of EMPOWER-UP while being interviewed to gain insight into their understanding of items and the mental processes needed to respond to them [21, 40, 41]. During interviews, we used a combination of the think aloud and concurrent verbal probing techniques [43, 44]. Participants were instructed to verbalise all thoughts as they responded to the items and informed that the interviewer might follow up with a combination of pre-determined probing questions and questions emerging during the interview [41, 44]. The interviews were conducted by EM (*n* = 21) and SB (*n* = 8) in person, via video calls, or by telephone as participants preferred. In-person interviews were conducted at hospital facilities or participants’ homes. All interviews were audio-recorded and transcribed.

#### Cross translation procedure

Cross translation followed international recommendations [45]. In the first step, two independent native English-speaking external translators translated the questionnaire into English. Disagreements were resolved through harmonisation by the translators and VZ and EM. Two independent native Danish-speaking external translators then translated the questionnaire back to Danish, followed by harmonisation involving all translators, VZ, and EM, to reach agreement on the final wording of the Danish and English versions.

Only cross-translated items were presented to participants during cognitive interviews, but preliminary item wordings were translated from Danish to English by a single translator for presentation here to allow readers to review items discarded during expert and cognitive evaluation (Fig. S2).

#### Data analysis

Data included transcribed cognitive interviews, interviewer notes, and audio recordings [46], which were all compared by EM after each interview and discussed with SB as needed to uncertainties. EM simultaneously conducted an informal analysis using text summary [46] to evaluate the questionnaire item by item. EM and VZ, and occasionally SB, discussed identified problems with specific items, and EM and VZ mutually agreed on solutions. Items were rejected or modified if two or more participants experienced difficulty understanding or answering them or based on convincing arguments from participants. If the best solution to an identified problem was unclear or only one participant expressed a specific concern without offering a convincing argument, items were retained and further evaluated in subsequent interview rounds. LL was consulted for methodological questions.

## Results

### Domains and items

Conceptual review of foundational research data generated 139 preliminary items: 30 from GT I [6], 17 from GT II [16], 15 from GT III [17], 29 from a qualitative evaluation of Guided Self-Determination [18], 10 from GT IV [32, 33], and 38 from the code book created during GT I-III analyses. Each item had a 7-point Likert-type response scale. Constant comparison reduced the preliminary pool to 46 items (Fig. S2, p1-p46) that covered the relational patterns explained in the grounded theories, distributed across four hypothesized domains corresponding to each theory. VZ and EM refined item wording by discussion to ensure the potential for generic use.

### Expert panel evaluation

As a result of the expert panel evaluation (Fig. S2, evaluation round E), the response scale was decreased to five options because panel members agreed it would be easier to distinguish between fewer response categories. Evaluation of the 46 preliminary items resulted in modified wording of 28 items (Fig. S2, filled squares), rejection of 11 items (Fig. S2, filled circles), acceptance of seven items (Fig. S2, p3, p5, p16, p22, p38, p40, p43) with no changes, and generation of six new items (Fig. S2, p47-p52).

Expert panel feedback expanded our understanding of what was required for broad generic use of items. For example, preliminary item p28, “I avoided going to appointments because I didn’t feel they benefitted me”, was intended to reflect resistance evidenced by non-attendance in outpatient clinics among healthcare users dissatisfied with HCPs [6]. However, a panel member pointed out that “going to appointments” is meaningless in home care. We realized that the wording also needed modification for users receiving care in a hospital ward. Consequently, we amended it to “I avoided interacting with the healthcare professional(s) because I didn’t feel it was good for me”, which reflected the theoretical concept of resistance. Our understanding similarly expanded for items p13 and p26, which pertained to prescription of new medication. Several panel members pointed that these items would not apply to all healthcare users. We rejected both and replaced them with a new, p50, “The healthcare professional(s) did not ask whether I was ready to follow their advice/prescriptions”, which reflected the concept of provider dominance in the original items [16].

Most wording modifications were intended to make items more easily understandable and eliminate specific sources of misunderstanding. As Fig. S2 depicts, modifications often occurred in several steps, starting in the expert panel but seldom finishing there. For example, the phrase, “I could easily express views that were different from those of the healthcare professional(s)”, in item p2 was expanded to include “about my situation” to clarify that the views in question pertained only to healthcare users’ circumstances. We similarly clarified item p4, “I felt involved in the care process in a meaningful way”, by replacing “the care process” with “decisions” to ensure it would be understood as concerned with decision-making processes.

Among rejected items, panel members agreed that the content of ten (Fig. S2, p8-9, p11, p13, p15, p18, p26, p30, p37, p42) would be adequately covered by some of the remaining items, while item p33 was rejected because a panel member pointed out that it reflected resistance against disease but no interaction with an HCP. Five (Fig. S2, p47-49, p51-52) of six new items were constructed to represent the theoretical concepts more comprehensively, and item p50 was generated to merge two other items, as noted.

After expert panel evaluation, 41 revised items were ready for further evaluation by healthcare users.

### Cognitive evaluation

Cognitive interviews [40, 41] on the Danish version were initially conducted with 26 native Danish-speaking participants through eight interviewing rounds, followed by two rounds with three native English-speaking participants on the cross-translated English version (Table 2). Each round evaluated a revised version of the questionnaire, beginning with the 41 items remaining after expert panel input. Twelve participants lived with diabetes, nine with cancer, and eight with schizophrenia, and participants were aged 28 to 78 years (mean, 55 years). All Danish interviews were conducted by telephone (*n* = 15) or in person (*n* = 11), while all English interviews were conducted by video calls. Most (*n* = 22) participants evaluated interactions with HCPs in outpatient clinics; seven evaluated interactions in hospital wards (*n* = 3), general practice (*n* = 2), and their homes (*n* = 2) (Table 2).

**Table 2:** Characteristics of healthcare users participating in cognitive interviews

Participant characteristics	Interview round										Total
	**1**	**2**	**3**	**4**	**5**	**6**	**7**	**8**	**9**	**10**	
**Number of participants**	2	5	3	7	2	2	2	3	2	1	29
**Female**	1	3	3	2	0	0	0	0	1	1	11
**Male**	1	2	0	5	2	2	2	3	1	0	18
**Disease group**											
**Diabetes**	1	1	0	5	0	2	0	0	2	1	12
**Cancer**	0	2	2	2	2	0	1	0	0	0	9
**Schizophrenia**	1	2	1	0	0	0	1	3	0	0	8
**Interviewing method**											
**Telephone**	0	1	1	5	1	2	2	3	1	0	16
**Video calls**	0	0	0	0	0	0	0	0	1	1	2
**Face-to-face**	2	4	2	2	1	0	0	0	0	0	11
**Settings evaluated**											
**Outpatient clinics**	2	5	1	6	2	2	0	2	1	1	22
**Inpatient hospital wards**	0	0	1	1	0	0	1	0	0	0	3
**General practice**	0	0	0	0	0	0	1	0	1	0	2
**Own home**	0	0	1	0	0	0	0	1	0	0	2

Interview rounds 1–8 with Danish-speaking participants and rounds 9–10 with English-speaking participants

#### Danish evaluation

During cognitive interviews with Danish-speaking participants (Fig. S2, rounds 1–8), we accepted seven items (p3, p6, p16, p22, p31, p44, p52), modified 21 (filled squares), rejected 13 (filled circles), and generated three new ones (p53-55). Four rejected items (p17, p23, p41, p48) were later re-introduced with revisions, as was one item (p9) rejected by the expert panel. In addition to revising items based on cognitive interview analyses, we also made three overall revisions based on insights we gained during the expert panel and interviews. Our primary overall revision—from negative to positive wording – is indicated with an asterisk before the final draft version in Fig. S2. The many negatively worded preliminary items were consistent with the viewpoint of GT I-III study participants, who had struggled for years with glucose levels above recommended targets, conflicts with HCPs, and lack of effective support. However, some cognitive interview participants voiced concerns about negative wording. In particular, one participant in the fourth round – with primarily positive experiences with the healthcare system—expressed intense scepticism about negative wording. This participant found it difficult to imagine any healthcare users having such negative experiences and perceived the entire questionnaire biased against the healthcare system. After consulting with LL, we decided to carefully review all items and consider balancing positive and negative wording. We consequently revised 10 items (p12, p14, p21, p23, p24, p29, p32, p39-41) to positive wording. However, some participants had described negative wording as legitimizing their negative experiences; we retained it in four items (p16, p22, p28, p31) where we believed it was especially important to reflect users’ negative experiences.

Two additional overall changes are not depicted in Fig. S2. All wording was changed from plural to singular in recognition that it was difficult for participants to evaluate more than one person at a time, and the composite *situation/condition/illness* term replaced various uses of the component words.

In terms of items accepted without changes, the interviewer probed for more information on several occasions to ensure that participants understood the items as intended. For example, participants were asked to explain in their own words what the items were asking or to describe a situation in which they had experienced the phenomenon the item represented.

Seven items (p1, p4, p12, p21, p24, p32, p40, p54) required only a single change, usually modifying or adding a word. For example, in item p1, “I could talk honestly about my difficulties with managing my situation/condition/illness”, “honestly” was replaced with “openly” because some participants stressed that the issue was not their ability to be honest. The issue was whether an interaction with an HCP was conducive to open and honest communication.

In most cases, items were revised multiple times Fig. S2. The remaining preliminary items included in the final draft were revised two (p19, p20, p28, p35, p39, p53), three (p2, p14, p29, p38, p45, p46), four (p5), five (p51), or six times (p49) during Danish interviewing rounds. Of the five re-introduced items, two (p9, p41) required no revisions, but we revised the other three items (p23), two (p17), and three (48) times. Some of these revisions were minor, but more extensive revisions were sometimes needed to ensure that theoretical concepts were adequately captured.

In particular, the concept of a *blurred sympathy relationship type* [16] proved challenging to capture. However, it was an important concept representing how HCPs could respond when they found it challenging to acknowledge problems that the healthcare users disclosed. This often led healthcare users to feel let down. For example, HCPs might exaggerate similarities with healthcare users by saying, for instance, “I have tried that myself, so I understand”, but it was obvious to users that their experiences were not similar. A male participant exemplified the difficulty of capturing this phenomenon. His questionnaire responses demonstrated strong disagreement with items reflecting blurred sympathy, indicating an empowering interaction, but his interview-statements revealed that he clearly perceived the interaction as disempowering. This finding led us to revise existing items intended to cover the concept and re-introduce revised versions of p17 and p48. The difficulty of capturing this concept was underscored by the need to revise items multiple times. Blurred sympathy can be characterized by warm and talkative interactions that remain superficial and unhelpful. Item p49, “The interaction had the character of something friendly”, had been unsuccessfully revised multiple times because participants continued to interpret it as reflecting a positive characteristic. Finally, “The interaction seemed too superficial to have any significance for my situation/condition/illness”, captured the essence of blurred sympathy.

The intended meaning of several items was unclear to users, leading to misinterpretations that prompted major revisions and exemplified the importance of including healthcare users diagnosed with schizophrenia in the evaluation. For example, one participant misunderstood the Danish wording of p35, “I got to explain in my own words what is difficult or challenging for me”, as reflecting the extent to which the HCP used the participant’s words to describe current challenges. Although the participant could not explain what part of the wording gave rise to this misunderstanding, another participant with schizophrenia in a subsequent interview round suggested that “in my own words” might be understood very literally by some individuals with the same diagnosis. We followed this suggestion, revising the item to “There was room in the interaction for me to explain what was difficult or challenging for me”, that reflected our intension of asking to what extent HCPs encourage and support healthcare users in describing their challenges in their own words.

In other cases, more substantial revisions were needed because participants made us aware that an item contained assumptions. An example was p14,”I withdrew because the healthcare professional(s) spoke in general terms and not about my situation”, which some participants found difficult to answer. They had experienced withdrawing from interactions but not necessarily because the HCP had failed to address issues relevant to their situation. We reworded the item to, “Along the way, I almost lost the desire to cooperate” (later revising the wording to positive: “The interaction made me want to continue with that particular healthcare professional”).

We deleted items that participants felt were irrelevant (p7, p10, p23, p25, p34, p36, p41, p47, p48), difficult to answer (p17, p25, p47), or difficult to comprehend (p41, p48) or suggested that other items covered the same content (p10, p17, p27, p43, p50). We first revised items that participants had difficulty answering or comprehending. If the challenges persisted, we reviewed the items again and deleted them if we believed other items adequately covered the concepts.

Although we did not ask participants about their educational backgrounds, several spontaneously offered this information during interviews. Interestingly, participants reporting a higher educational background tended to underestimate the abilities of individuals with less education to comprehend some items. For instance, in evaluation round 2, a participant with a higher academic degree expressed concerns about p39, “Even though I told the healthcare professional(s) about something inappropriate I do, they didn’t clarify the reasons for it”. She said: “… something inappropriate.. well, I can imagine how that would be a difficult word for some… you know, like, with less education and such…”. We then asked probing questions about ‘inappropriate’ in two subsequent interviews with participants who reported having no formal education. They easily understood the intended meaning, emphasising the importance of including participants with varying backgrounds.

In addition to revising individual items, we also amended the questionnaire’s introductory text because many participants found it difficult to choose a response category if they felt an item did not apply to them. After discussion with LL, we added a sentence instructing participants to choose the ‘neither agree nor disagree’ response option in that case.

#### Translation

We obtained no further insights after round eight of cognitive evaluation in Danish no further insights were obtained and began cross translation. During the process, we made minor revisions to the wording of the introductory text and eight items (p2, p6, p21, p24, p46, p48, p49, p53), including exchanging some words for more colloquial language. In addition, we ensured that all items used the past tense and referred to the healthcare user before referring to the HCP.

#### English evaluation

After reaching agreement on both Danish and English wording, we interviewed three native English-speaking participants (Table 2) in the final two rounds. We accepted 27 items and modified ten Fig. S2. Among the latter, we revised nine items (p4, p20, p21, p23, p29, p32, p46, p48, p55) once and one items (p40) twice. We changed only the Danish (p4) or English (p23, p46) versions of three items and revised both versions of the remaining seven items.

We changed or added a single word to four items (p4, p23, p32, p48), as exemplified by replacing “strengths” with “strong sides” in item p23, “Through the interaction, the healthcare professional supported me in seeing my strengths”. We made more substantial revisions to the remaining five items, e.g., changing item 40 from “Through the interaction we managed to get to the heart of the matter” to “Through the interaction we managed to get to what I believe is the heart of the matter in my situation/condition/illness”. This change made it clear that the healthcare user determines what constitutes the heart of the matter.

## Discussion

EMPOWER-UP was developed to enable evaluating healthcare users’ perceptions of the empowering qualities of their interactions with HCPs and is suggested as part of the solution to an almost 40-year failure to translate empowerment into practice [10, 11]. The initial development and validation stages were indispensable to ensuring the face and content validity of EMPOWER-UP and its ability to meet the first three criteria noted in the introduction to this article: 1) focus on barriers and enablers to empowerment in decision-making and problem-solving processes, 2) draw on knowledge from a rigorous qualitative conceptual foundation derived from grounded theory, and 3) applicable for use across the healthcare system regardless of LTHCs, healthcare settings, and HCPs’ professional background. Using expert panel and cognitive interviews, we aligned EMPOWER-UP with these criteria and reduced the preliminary pool of 139 items to the 36-item draft Danish and English versions of the questionnaire. The English draft version can be found in Additional file 1.

Although numerous questionnaires have been developed to assess the shared decision-making process, a recent systematic review found that most had fatal quality flaws and were poorly validated [47]. Moreover, these instruments assess decision-making processes but not relational problem-solving. The selection of the domain of empowerment during relational decision-making and problem-solving for EMPOWER-UP is justified by the multitudinous occurrences of both activities across the healthcare system, constituting both an enormous potential source of empowerment and a high risk of disempowerment. Importantly, development of EMPOWER-UP was based on a theoretical framework comprising extensive knowledge of empowerment enablers and barriers. It explains the changes that occur when these barriers are overcome [18, 33], underscoring the potential utility of EMPOWER-UP to measure them.

The initial three grounded theories [6, 16, 17] involved participants sharing frustration about a healthcare system seemingly unable to help them change their situations. This resulted in many negatively worded quotations and, thus, preliminary items. Although we consider it important to include and validate such experiences, our results demonstrate the importance of also including healthcare users with positive experiences. Some participants were sceptical about the negative items and perceived the questionnaire is biased against the healthcare system, and a more well-balanced distribution between positive and negative items may ensure broader applicability of the questionnaire.

One of the major strengths of EMPOWER-UP is the systematic development process building on a foundation of four interrelated grounded theories based on both healthcare user and professional perceptions and observations of interprofessional discussions. The recommendation of using grounded theory to ensure content validity [26, 48, 49] is likely connected with the constant comparison method, which ensures that concepts are meticulously developed to unravel the essence of the studied interactions [24]. As we initially reduced the number of preliminary questionnaire items, we benefitted from the foundation of clearly defined and interconnected concepts in the grounded theories, in conjunction with statements and observations from users and providers of healthcare in usual care [6, 16, 17] and in care improved by an empowerment-based intervention [18, 33]. In formulating and reformulating items, we also used the constant comparison method [32] from grounded theory to capture the essence of the phenomena. This method helped us move back and forth between preliminary items, theoretical concepts, and quotes to ensure coverage of the most important concepts.

The four foundational grounded theories explain power structures in user-provider interactions across healthcare that are usually hidden. The need for knowledge of such structures to overcome barriers to empowerment was recognized in Canadian health promotion more than 20 years ago [5], highlighting the importance of our endeavours to include this knowledge in the EMPOWER-UP questionnaire. Moreover, the cognitive interviews allowed us to ensure that the questionnaire adequately covers the complex theoretical concept of a blurred sympathy relationship type [16]. Not surprisingly, this concept proved difficult to capture and its complexity arises from the fact that this kind of relationship may initially be perceived as pleasant because it is characterized by HCP attempts dispel tension equating themselves with the healthcare user [16]. Such interactions can be misinterpreted as signalling compassion, but the constant comparison in the grounded theory-analyses revealed the absence of an empowering potential for change [16], evidenced by healthcare users feeling let down due to the lack of support after sharing difficult feelings with HCPs. Users may perceive these HCPs as nice and easy to talk to but ultimately less than helpful, as exemplified by one participant from GT II: “We have really talked about many different things, but in relation to my treatment it hasn’t made any difference” ([24], p.632]). Thus, the empowering qualities of decision-making and problem-solving processes lie not in whether healthcare users perceive HCPs as nice, but in whether HCPs can establish a constructive partnership and take users seriously [16]. This point was raised by Paterson [50] when a participant described a HCP able to foster such partnerships as “crusty, no sense of humour, very matter of fact, but willing to really hear me when I say what I think is happening and what needs to happen” (p. 577).

An increasing number of healthcare users live with more than one LTHC, representing a major health challenge [51] that reduces quality of life [52]. The availability of questionnaires that can be used across conditions is thus increasingly important, reflected in a larger trend toward developing generic questionnaires on empowerment [3]. Such questionnaires may help identify appropriate interventions to support users across the healthcare system in developing condition-specific life skills [34–36]. However, the wording in several of these questionnaires remain limited to specific HCPs, i.e., doctors [53] or nurses [54], or to health conditions requiring medication [55]. In addition to an intense focus on ensuring the use of generic wording in the early stages of item development, our expert panel provided invaluable feedback to ensure EMPOWER-UP’s potential for generic use across settings and for LTHCs not requiring medical treatments.

Individuals with schizophrenia may experience barriers to participating in research and decision-making in healthcare. This involves being perceived as someone who cannot autonomously decide on participation or provide informed consent [56]. It may also entail struggles concerning not being seen as a competent person with valuable experiences [57]. In this study, we deliberately included these individuals to ensure the applicability of EMPOWER-UP in settings involving healthcare users who are often described as vulnerable or with limited cognitive abilities. During the interviews, we found that these participants were equally capable of providing useful feedback on the wording and comprehension of questionnaire items compared to participants diagnosed with cancer or diabetes. This ability to provide important contributions through qualitative interviews aligns with the experiences of Newman et al. [58]. The most significant difference between our three participant groups was that individuals with cancer or diabetes seemed more accustomed to having their perceptions and opinions valued. In contrast, participants with schizophrenia expressed more gratitude for being allowed to contribute. Accordingly, including healthcare users with the three highly varied and complex conditions of diabetes, cancer, and serious mental illness in cognitive interviews substantially supports the generic application of EMPOWER-UP helping to ensure that its use is meaningful for healthcare users with serious physical and psychiatric conditions.

A related strength of EMPOWER-UP is the meticulously developed item wording supporting its generic use in clinical practice and research across healthcare. Although the questionnaire’s conceptual framework was originally developed in the context of diabetes [32], the theoretical framework has been recognised in various other contexts. The wide dissemination of an empowerment-based intervention based on the same framework [19, 37–39] suggests that more similarities than differences exist between users with various LTHCs in terms of their sparse experiences of realized empowerment and strengthens the argument that a questionnaire built on these patterns is applicable across healthcare. In future attempts to close the gap between the aim of empowerment and its realization in practice, clinicians and researchers must be able to compare and contrast promising interventions, providing decision-makers knowledge about which interventions are most useful for whom and where.

### Limitations

Several limitations deserve mention. We did not search the literature for additional grounded theories and cannot rule out the possibility that other existing theories might have been useful in developing EMPOWER-UP. However, the patterns explained in our theoretical foundation had proved applicable across 12 heterogeneous conditions and settings, and we consider this an adequately thorough theoretical foundation for the questionnaire. It is unlikely that other theories would have significantly changed the development of the questionnaire.

Empowerment has long been recommended in long-term care, and an increasing awareness of the need to translate empowerment into acute and critical care has emerged [59]. The investigation reported here precludes conclusions about the usefulness of EMPOWER-UP in these contexts, and further research is warranted. The decision to exclude items before the expert panel and cognitive evaluation may be considered a potential limitation. However, in light of the difficulty for participants in the expert panel and cognitive interviews of providing accurate evaluations of the 139 original preliminary items and VZ’s extensive knowledge of the conceptual foundation as the developer of all four grounded theories, we considered this a reasonable choice. Finally, our choice to end the cognitive interviewing in English after two rounds including a total of three participants with diabetes may be considered a limitation. This choice arose primarily from the difficulties of recruiting participants during the Covid-19 pandemic. However, because no major difficulties in item comprehension emerged in the final interviewing round, we considered it acceptable to end the evaluation. Translating the questionnaire into other languages or using it in other contexts would benefit from further cognitive evaluation [60].

## Conclusions

This article reports on the development and content and face validation of EMPOWER-UP, designed as a generic measure to evaluate empowerment in decision-making and problem-solving processes as perceived by healthcare users. The questionnaire was initially developed in Danish and subsequently translated to English. Content and face validation resulted in a 36-item draft questionnaire with the potential for broad application across LTHCs, healthcare settings, and HCP professional backgrounds.

In a coming article, we aim to present the next steps in the development and validation process, namely the examination of the questionnaire’s psychometric properties among a sample of up to 720 individuals diagnosed with diabetes, cancer, and mental illness. This will comprise a reporting of the results of factor analysis; item response theory modelling, including differential item functioning analysis; and an evaluation of the final questionnaire’s internal consistency and reliability.

## Supplementary Information

Additional file 1.

Additional file 2. Figure S2. Items evaluated by the expert panel and healthcare users in cognitive interviews. Note: Squares represent modifications to item content, circles represent item elimination, and an asterisk represents negative-to-positive modification to final draft wording. Final draft items (d1-36) were cross translated into English after round 8 of Danish cognitive interviews. For publication purposes only, all preliminary items were translated into English by a single translator. Abbreviations: E, expert panel, T, translation. 

## Data Availability

The original wording of all versions of the Danish and English items are available upon reasonable request, as are items that were eliminated before expert panel evaluation. The data may be obtained by contacting the corresponding author via email: emilie.haarslev.schröder.marqvorsen@regionh.dk.

## References

[1] Aujoulat I, D’Hoore W, Deccache A. Patient empowerment in theory and practice: Polysemy or cacophony? Patient Educ Couns. 2007;66(1):13–20. 10.1016/j.pec.2006.09.008. 17084059 10.1016/j.pec.2006.09.008

[2] Anderson RM, Funnell MM. Patient empowerment: Myths and misconceptions. Patient Educ Couns. 2010;79(3):277–82. 10.1016/j.pec.2009.07.025. 19682830 10.1016/j.pec.2009.07.025 PMC2879465

[3] Mora MA, Sparud-Lundin C, Moons P, Bratt E-L. Definitions, instruments and correlates of patient empowerment: a descriptive review. Patient Educ Couns. 2022;105(2):346–55. 10.1016/j.pec.2021.06.014. 34140196 10.1016/j.pec.2021.06.014

[4] Funnell MM, Anderson RM, Arnold MS, Barr PA, Donnelly M, Johnson PD, et al. Empowerment: an idea whose time has come in diabetes education. Diabetes Educ. 1991;17(1):37–41. 10.1177/014572179101700108. 1986902 10.1177/014572179101700108

[5] VanderPlaat M. Locating the feminist scholar: relational empowerment and social activism. Qual Health Res. 1999;9(6):773–85. 10.1177/104973299129122270. 10662258 10.1177/104973299129122270

[6] Zoffmann V, Kirkevold M. Life versus disease in difficult diabetes care: conflicting perspectives disempower patients and professionals in problem solving. Qual Health Res. 2005;15(6):750–65. 10.1177/1049732304273888. 15961873 10.1177/1049732304273888

[7] Castro EM, Van Regenmortel T, Vanhaecht K, Sermeus W, Van Hecke A. Patient empowerment, patient participation and patient-centeredness in hospital care: a concept analysis based on a literature review. Patient Educ Couns. 2016;99(12):1923–39. 10.1016/j.pec.2016.07.026. 27450481 10.1016/j.pec.2016.07.026

[8] Wallerstein N, Bernstein E. Empowerment education: Freire’s ideas adapted to health education. Heal Educ Quaterly. 1988;15(4):379–94. 10.1177/109019818801500402. 10.1177/109019818801500402 3230016

[9] Werbrouck A, Swinnen E, Kerckhofs E, Buyl R, Beckwée D, De Wit L. How to empower patients? A systematic review and meta-analysis. Transl Behav Med. 2018;8(5):660–74. 10.1093/tbm/iby064. 29982675 10.1093/tbm/iby064

[10] Miller T, Reihlen M. Assessing the impact of patient-involvement healthcare strategies on patients, providers, and the healthcare system: a systematic review. Patient Educ Couns. 2023;110:107652. 10.1016/j.pec.2023.107652. 36804578 10.1016/j.pec.2023.107652

[11] World Health Organization. Promoting health in the SDGs: report on the 9th global conference for health promotion. Shanghai; 2017. Available from: https://www.who.int/publications/i/item/promoting-health-in-the-sdgs. Accessed 17 Nov 2023

[12] Halvorsen K, Dihle A, Hansen C, Nordhaug M, Jerpseth H, Tveiten S, et al. Empowerment in healthcare: a thematic synthesis and critical discussion of concept analyses of empowerment. Patient Educ Couns. 2020;103(7):1263–71. 10.1016/J.PEC.2020.02.017. 32164960 10.1016/j.pec.2020.02.017

[13] Ekman I, Swedberg K, Taft C, Lindseth A, Norberg A, Brink E, et al. Person-centered care - ready for prime time. Eur J Cardiovasc Nurs. 2011;10(4):248–51. 10.1016/J.EJCNURSE.2011.06.008. 21764386 10.1016/j.ejcnurse.2011.06.008

[14] Havana T, Kuha S, Laukka E, Kanste O. Patients’ experiences of patient-centred care in hospital setting: a systematic review of qualitative studies. Scand J Caring Sci. 2023;27(4):1001. 10.1111/SCS.13174. 10.1111/scs.13174 37066838

[15] Didier A, Nathaniel A, Scott H, Look S, Benaroyo L, Zumstein-Shaha M. Protecting personhood: a classic grounded theory. Qual Health Res. 2023;33(13):1177–88. 10.1177/10497323231190329. 37669352 10.1177/10497323231190329 PMC10626982

[16] Zoffmann V, Kirkevold M. Relationships and their potential for change developed in difficult type 1 diabetes. Qual Health Res. 2007;17(5):625–38. 10.1177/1049732307301230. 17478645 10.1177/1049732307301230

[17] Zoffmann V, Harder I, Kirkevold M. A person-centered communication and reflection model: sharing decision-making in chronic care. Qual Health Res. 2008;18(5):670–85. 10.1177/1049732307311008. 18223158 10.1177/1049732307311008

[18] Zoffmann V, Kirkevold M. Realizing empowerment in difficult diabetes care: a guided self-determination intervention. Qual Health Res. 2012;22(1):103–18. 10.1177/1049732311420735. 21876206 10.1177/1049732311420735

[19] Olesen ML, Jørgensen R. Impact of the person-centred intervention guided self-determination across healthcare settings - an integrated review. Scand J Caring Sci. 2022;00:1–23. 10.1111/SCS.13138. 10.1111/scs.13138 36524250

[20] Fayers PM, Machin D. Quality of Life. 3rd ed. Chichester, West Sussex: Wiley Blackwell; 2016.

[21] Boateng GO, Neilands TB, Frongillo EA, Melgar-Quiñonez HR, Young SL. Best practices for developing and validating scales for health, social, and behavioral research: a primer. Front Public Heal. 2018;6:149. 10.3389/fpubh.2018.00149. 10.3389/fpubh.2018.00149 PMC6004510 29942800

[22] de Vet HC, Terwee CB, Mokkink LB, Knol DL. Measurement in medicine. Cambridge: Cambridge University Press; 2011.

[23] Strauss ME, Smith GT. Construct validity: advances in theory and methodology. Annu Rev Clin Psychol. 2009;5:1–25. 10.1146/annurev.clinpsy.032408.153639. 19086835 10.1146/annurev.clinpsy.032408.153639 PMC2739261

[24] Glaser B. Theoretical sensitivity. San Francisco: University of California; 1978.

[25] Cheng KKF, Clark AM. Qualitative methods and patient-reported outcomes: measures development and adaptation. Int J Qual Methods. 2017;16:1–3. 10.1177/1609406917702983.

[26] Lasch K, Marquis P, Vigneux M, Abetz L, Arnould B, Bayliss M, et al. PRO development: rigorous qualitative research as the crucial foundation. Qual Life Res. 2010;19(8):1087–96. 10.1007/s11136-010-9677-6. 20512662 10.1007/s11136-010-9677-6 PMC2940042

[27] Pekonen A, Eloranta S, Stolt M, Virolainen P, Leino-Kilpi H. Measuring patient empowerment – A systematic review. Patient Educ Couns. 2019;103:777. 10.1016/J.PEC.2019.10.019. 31767243 10.1016/j.pec.2019.10.019

[28] Terwee CB, Prinsen CAC, Chiarotto A, Westerman MJ, Patrick DL, Alonso J, et al. COSMIN methodology for evaluating the content validity of patient-reported outcome measures: a Delphi study. Qual Life Res. 2018;27(5):1159–70. 10.1007/S11136-018-1829-0. 29550964 10.1007/s11136-018-1829-0 PMC5891557

[29] Mokkink LB, Terwee CB, Patrick DL, Alonso J, Stratford PW, Knol DL, et al. The COSMIN study reached international consensus on taxonomy, terminology, and definitions of measurement properties for health-related patient-reported outcomes. J Clin Epidemiol. 2010;63(7):737–45. 10.1016/j.jclinepi.2010.02.006. 20494804 10.1016/j.jclinepi.2010.02.006

[30] Glasgow RE. What does it mean to be pragmatic? Pragmatic methods, measures, and models to facilitate research translation. Health Educ J. 2013;40(3):257–65. 10.1177/1090198113486805. 10.1177/1090198113486805 23709579

[31] Strübing J. Research as pragmatic problem solving: the pragmatist roots of empirically-grounded theorizing. In: Bryant T, Charmaz A, editors. *SAGE Handb grounded theory*. SAGE Publications Ltd; 2007. p. 552–602. 10.4135/9781848607941

[32] Zoffmann V. Guided self-determination, a life skills approach developed in difficult type 1 diabetes, PhD thesis. 1. edition. [Århus]. Aarhus C: Department of Nursing Science, University of Aarhus; 2004.

[33] Zoffmann V, Jørgensen R, Graue M, Biener SN, Brorsson AL, Christiansen CH, et al. Person-specific evidence has the ability to mobilize relational capacity: a four-step grounded theory developed in people with long-term health conditions. Nurs Inq. 2023;30(3):e12555. 10.1111/NIN.12555. 37062853 10.1111/nin.12555

[34] Allen S, Mehal M, Palmateer S, Sluser R, editors. The new dynamics of life skills coaching. Toronto: YWCA; 1995.

[35] Nutbeam D. Health promotion glossary. Heal Promot. 1986;13(4):349–65. 10.1093/HEAPRO/1.1.113. 10.1093/heapro/1.1.113 10318625

[36] Mullen D. A conceptual framework for the life skills program. Ottawa: Minister of Supply and Services; 1985.

[37] Olesen ML, Rossen S, Jørgensen R, Langballe Udbjørg L, Hansson H. Usefulness of a Ddgitally assisted person-centered care intervention: qualitative study of patients’ and nurses’ experiences in a long-term perspective. JMIR Nurs. 2023;6:e46673. 10.2196/46673. 37200076 10.2196/46673 PMC10236280

[38] Dehn P, Munch Simonsen S, Olesen ML. Multidimensional factors determine skill acquisition development in guided self-determination: a qualitative study. Scand J Caring Sci. 2023;37(2):549–60. 10.1111/scs.13140. 36533327 10.1111/scs.13140

[39] Rasmussen B, Wynter K, Hamblin PS, Rodda C, Steele C, Holton S, et al. Feasibility and acceptability of an online guided self-determination program to improve diabetes self-management in young adults. Digit Heal. 2023;9:20552076231167010. 10.1177/20552076231167008. 10.1177/20552076231167008 PMC10068990 37021125

[40] Beatty PC, Willis GB. Research synthesis: the practice of cognitive interviewing. Public Opin Q. 2007;71(2):287–311. 10.1093/poq/nfm006.

[41] Meadows K. Cognitive interviewing methodologies. Clin Nurs Res. 2021;30(4):375–9. 10.1177/10547738211014099. 33998325 10.1177/10547738211014099

[42] Epstein J, Santo RM, Guillemin F. A review of guidelines for cross-cultural adaptation of questionnaires could not bring out a consensus. J Clin Epidemiol. 2015;68(4):435–41. 10.1016/j.jclinepi.2014.11.021. 25698408 10.1016/j.jclinepi.2014.11.021

[43] Willis GB. Cognitive interviewing : a tool for improving questionnaire design. Thousand Oaks, CA: Sage; 2005.

[44] Ryan K, Gannon-Slater N, Culbertson MJ. Improving survey methods with cognitive interviews in small- and medium-scale evaluations. Am J Eval. 2012;33(3):414–30. 10.1177/1098214012441499.

[45] Wild D, Grove A, Martin M, Eremenco S, McElroy S, Verjee-Lorenz A, et al. Principles of good practice for the translation and cultural adaptation process for patient-reported outcomes (PRO) measures: report of the ISPOR Task Force for Translation and Cultural Adaptation. Value Heal. 2005;8(2):94–104. 10.1111/j.1524-4733.2005.04054.x. 10.1111/j.1524-4733.2005.04054.x 15804318

[46] Willis GB. Analysis of the cognitive interview in questionnaire design. Oxford: Oxford University Press; 2015.

[47] Ahmad M, Abu Tabar N, Othman EH, Abdelrahim Z. Shared decision-making measures: a systematic review. Qual Manag Health Care. 2020;29(2):54–66. 10.1097/QMH.0000000000000250. 32224789 10.1097/QMH.0000000000000250

[48] Brod M, Tesler LE, Christensen TL. Qualitative research and content validity: developing best practices based on science and experience. Qual Life Res. 2009;18(9):1263–78. 10.1007/S11136-009-9540-9. 19784865 10.1007/s11136-009-9540-9

[49] Patrick DL, Burke LB, Gwaltney CJ, Leidy NK, Martin ML, Molsen E, et al. Content validity - establishing and reporting the evidence in newly developed patient-reported outcomes (PRO) instruments for medical product evaluation: ISPOR PRO Good Research Practices Task Force Report: part 1 - eliciting concepts for a new PRO instru. Value Heal. 2011;14(8):967–77. 10.1016/J.JVAL.2011.06.014. 10.1016/j.jval.2011.06.014 22152165

[50] Paterson B. Myth of empowerment in chronic illness. J Adv Nurs. 2001;34(5):574–81. 10.1046/j.1365-2648.2001.01786.x. 11380725 10.1046/j.1365-2648.2001.01786.x

[51] World Health Organization. Noncommunicable diseases. 2023. Available from: https://www.who.int/news-room/fact-sheets/detail/noncommunicable-diseases. Accessed 19 May 2024

[52] Makovski TT, Schmitz S, Zeegers MP, Stranges S, van den Akker M. Multimorbidity and quality of life: systematic literature review and meta-analysis. Ageing Res Rev. 2019;53:100903. 10.1016/j.arr.2019.04.005. 31048032 10.1016/j.arr.2019.04.005

[53] Small N, Bower P, Chew-Graham CA, Whalley D, Protheroe J. Patient empowerment in long-term conditions: development and preliminary testing of a new measure. BMC Health Serv Res. 2013;13:263. 10.1186/1472-6963-13-263. 23835131 10.1186/1472-6963-13-263 PMC3725177

[54] Faulkner M. A measure of patient empowerment in hospital environments catering for older people. J Adv Nurs. 2001;34(5):676–86. 10.1046/j.1365-2648.2001.01797.x. 11380736 10.1046/j.1365-2648.2001.01797.x

[55] Zhou C, Ji X, Tan J, Wu Y. Psychometric properties of the Chinese version of the client empowerment scale in chronic patients. Springerplus. 2016;5:1636. 10.1186/s40064-016-3183-4. 27722054 10.1186/s40064-016-3183-4 PMC5031582

[56] Koivisto K, Janhonen S, Latvala E, Väisänen L. Applying ethical guidelines in nursing research on people with mental illness. Nurs Ethics. 2001;8(4):328–39. 10.1177/096973300100800405. 16004087 10.1177/096973300100800405

[57] Dahlqvist Jönsson P, Schön U-K, Rosenberg D, Sandlund M, Svedberg P. Service users’ experiences of participation in decision making in mental health services. J Psychiatr Ment Health Nurs. 2015;22(9):688–97. 10.1111/jpm.12246. 26148016 10.1111/jpm.12246

[58] Newman D, O’Reilly P, Lee SH, Kennedy C. Challenges in accessing and interviewing participants with severe mental illness. Nurse Res. 2017;25(1):37–42. 10.7748/nr.2017.e1443. 28639528 10.7748/nr.2017.e1443

[59] Wåhlin I. Empowerment in critical care - a concept analysis. Scand J Caring Sci. 2017;31:164–74. 10.1111/scs.12331. 27164009 10.1111/scs.12331

[60] Leidy NK, Vernon M. Perspectives on patient-reported outcomes: content validity and qualitative research in a changing clinical trial environment. Pharmacoeconomics. 2008;26(5):363–70. 10.2165/00019053-200826050-00002. 18429654 10.2165/00019053-200826050-00002

[61] World Medical Association. World Medical Association Declaration of Helsinki: ethical principles for medical research involving human subjects. JAMA. 2013;310(20):2191–4. 10.1001/jama.2013.281053. 10.1001/jama.2013.281053 24141714

