# Supplementary material for: Face and content validity of the EMPOWER-UP questionnaire: a generic measure of empowerment in relational decision-making and problem-solving
Source: BMC Med Inform Decis Mak. 2024 Oct 28;24:313. doi: 10.1186/s12911-024-02727-5 (PMC11514851; doi:10.1186/s12911-024-02727-5)
Supplement: Supplementary file 1 — Additional file 1. [file 12911_2024_2727_MOESM1_ESM.docx]

**EMPOWER-UP**

Research has shown that the interaction with healthcare professionals can have great significance for whether people are empowered to manage long-term health problems.

This questionnaire asks not only whether the healthcare professionals are friendly, but whether they support you to manage the particular challenges you experience with the health problems in your everyday life.

The questionnaire contains a number of statements concerning **the interaction between you and the healthcare professional in the management of your situation/condition/illness.**

The statements all concern your perception of what happened, considering both your own reaction and that of the healthcare professional. We would like you to consider the statements below and use the scale on the right to indicate the extent to which you agree or disagree with each of them.

We ask you to mark the box corresponding to the answer you think is most appropriate. If you do not think that the statement is relevant to your particular case, you can mark the box corresponding to “Neither agree nor disagree”.

Your answers will be treated confidentially.

| Please, answer the questions concerning the most recent interaction you have had with a healthcare professional. |
| --- |

**My perception of the interaction with the healthcare professional is that:**

|  | Strongly disagree | Disagree | Neither agree nor disagree | Agree | Strongly agree |
| --- | --- | --- | --- | --- | --- |
| 1. I could talk openly about my difficulties with managing my situation/condition/illness |  |  |  |  |  |
| 2. The healthcare professional took me seriously |  |  |  |  |  |
| 3. The healthcare professional showed interest in my experience with my situation/condition/illness |  |  |  |  |  |
| 4. There was room in the interaction for me to explain what was difficult or challenging for me |  |  |  |  |  |
| 5. The interaction with the healthcare professional strengthened my understanding of my situation/condition/illness |  |  |  |  |  |
| 6. The healthcare professional asked what I think about my situation/condition/illness |  |  |  |  |  |
| 7. Through the interaction we managed to get to what I believe is the heart of the matter in my situation/condition/illness |  |  |  |  |  |
| 8. The healthcare professional and I reflected together on my handling of the situation/condition/illness |  |  |  |  |  |
| 9. I felt involved in important decisions about my situation/condition/illness |  |  |  |  |  |
| 10. There was focus on something that we both believed was important for me |  |  |  |  |  |
| 11. The healthcare professional gave me room to explain how it really is for me to live with the situation/condition/illness |  |  |  |  |  |
| 12. The healthcare professional mistakenly believed that s/he understood my situation/condition/illness based on their own experiences |  |  |  |  |  |
| 13. There was room for me to share my views on the situation/condition/illness even if they were different from the healthcare professional's |  |  |  |  |  |
| 14. Irrelevant small talk took up too much time during the interaction |  |  |  |  |  |
| 15. Both my experience of living with the situation/condition/illness and the healthcare professional’s professional knowledge influenced the decision about what to do |  |  |  |  |  |
| 16. The interaction made me want to continue with that particular healthcare professional |  |  |  |  |  |
| 17. During the interaction there was room to talk about difficult topics |  |  |  |  |  |
| 18. Through the interaction I became aware of some things I can do myself to manage my situation/condition/illness |  |  |  |  |  |
| 19. The interaction gave me a clear picture of the benefits I can get from managing my situation/condition/illness in an ideal way in everyday life |  |  |  |  |  |
| 20. During the interaction it was only the healthcare professional who decided what we should discuss |  |  |  |  |  |
| 21. I felt that the interaction with the healthcare professional was a waste of time |  |  |  |  |  |
| 22. The healthcare professional showed frustration towards me |  |  |  |  |  |
| 23. Through the interaction I got a valuable insight into the healthcare professional’s thoughts about my way of managing the situation/condition/illness |  |  |  |  |  |
| 24. The healthcare professional gave me something to think about in relation to my way of managing my situation/condition/illness |  |  |  |  |  |
| 25. During the interaction, the healthcare professional appeared to take-over all responsibility for my situation/condition/illness |  |  |  |  |  |
| 26. The healthcare professional seemed to have given up on me |  |  |  |  |  |
| 27. The healthcare professional strengthened my confidence that I can handle challenges in my situation/condition/illness in the future |  |  |  |  |  |
| 28. The interaction with the healthcare professional made me want to withdraw |  |  |  |  |  |
| 29. We identified some things that made it clear to me that I must act differently to manage my situation/condition/illness in future |  |  |  |  |  |
| 30. The healthcare professional recognized the importance of my efforts in managing the situation/condition/illness |  |  |  |  |  |
| 31. The interaction seemed too superficial to have any significance for my situation/condition/illness |  |  |  |  |  |
| 32. I received adequate support to make my life and my situation/condition/illness fit together |  |  |  |  |  |
| 33. I felt comfortable telling the healthcare professional if I, in my management of the situation/condition/illness, made different choices than agreed upon |  |  |  |  |  |
| 34. Through the interaction, the healthcare professional supported me in seeing my strong sides |  |  |  |  |  |
| 35. I felt that the healthcare professional used knowledge about me in a constructive way |  |  |  |  |  |
| 36. The interaction gave me a deeper insight into my way of managing the situation/condition/illness |  |  |  |  |  |
