# Supplementary material for: Face and content validity of the EMPOWER-UP questionnaire: a generic measure of empowerment in relational decision-making and problem-solving
Source: BMC Med Inform Decis Mak. 2024 Oct 28;24:313. doi: 10.1186/s12911-024-02727-5 (PMC11514851; doi:10.1186/s12911-024-02727-5)
Supplement: Supplementary file 2 — Additional file 2. Figure S2. Items evaluated by the expert panel and healthcare users in cognitive interviews. Note: Squares represent modifications to item content, circles represent item elimination, and an asterisk represents negative-to-positive modification to final draft wording. Final draft items (d1-36) were cross translated into English after round 8 of Danish cognitive interviews. For publication purposes only, all preliminary items were translated into English by a single translator. Abbreviations: E, expert panel, T, translation. [file 12911_2024_2727_MOESM2_ESM.docx]

| **Preliminary items evaluated in expert panel and cognitive interviews** | | **Evaluation round** | | | | | | | | | | | | **Revised draft items selected for No. psychometric testing** | |
| --- | --- | --- | --- | --- | --- | --- | --- | --- | --- | --- | --- | --- | --- | --- | --- |
|  |  | **E** | **1** | **2** | **3** | **4** | **5** | **6** | **7** | **8** | **T** | **9** | **10** |  |  |
| p1 | I could be honest about my difficulties with managing the situation/condition/illness |  |  |  |  |  |  |  |  |  |  |  |  | I could talk openly about my difficulties with managing my situation/condition/illness | d1 |
| p2 | I could easily express views that were different from those of the healthcare professional(s) |  |  |  |  |  |  |  |  |  |  |  |  | There was room for me to share my views on the situation/condition/illness even if they were different from the healthcare professional’s | d13 |
| p3 | The healthcare professional(s) took me seriously |  |  |  |  |  |  |  |  |  |  |  |  | The healthcare professional took me seriously | d2 |
| p4 | I felt involved in the care process in a meaningful way |  |  |  |  |  |  |  |  |  |  |  |  | I felt involved in important decisions about my situation/condition/illness | d9 |
| p5 | I was supported in taking responsibility for my situation |  |  |  |  |  |  |  |  |  |  |  |  | The healthcare professional recognized the importance of my efforts in managing the situation/condition/illness | d30 |
| p6 | We identified some things that made it clear to me that I must do something about my situation |  |  |  |  |  |  |  |  |  |  |  |  | We identified some things that made it clear to me that I must act differently to manage my situation/condition/illness in future | d29 |
| p7 | The interaction with the healthcare professional(s) has taught me what I should specifically do in difficult situations |  |  |  |  |  |  |  |  |  |  |  |  |  | - |
| p8 | I saw new opportunities in my way of managing the condition/situation/illness |  |  |  |  |  |  |  |  |  |  |  |  |  | - |
| p9 | The interaction with the healthcare professional(s) gave me a self-insight that could take me further |  |  |  |  |  |  |  |  |  |  |  |  | The interaction gave me a deeper insight into my way of managing the situation/condition/illness | d36 |
| p10 | I discovered some patterns that I could change |  |  |  |  |  |  |  |  |  |  |  |  |  | - |
| p11 | I felt sidelined because the healthcare professional(s) took over |  |  |  |  |  |  |  |  |  |  |  |  |  | - |
| p12 | The healthcare professional(s) did not consider my experiences with the condition/situation/illness |  |  |  |  |  |  |  |  |  |  |  |  | *The healthcare professional showed interest in my experience with my situation/condition/illness | d3 |
| p13 | I was prescribed medicine without being asked whether I was ready to take it*  * If you haven’t been prescribed medicine, mark here __ |  |  |  |  |  |  |  |  |  |  |  |  |  | - |
| p14 | I withdrew because the healthcare professional(s) spoke in general terms and not about my situation |  |  |  |  |  |  |  |  |  |  |  |  | *The interaction made me want to continue with that particular healthcare professional | d16 |
| p15 | I didn’t say no when the healthcare professional(s) took it for granted that I would follow their advice |  |  |  |  |  |  |  |  |  |  |  |  |  | - |
| p16 | I felt that the interaction with the healthcare professional(s) was a waste of time |  |  |  |  |  |  |  |  |  |  |  |  | I felt that the interaction with the healthcare professional was a waste of time | d21 |
| p17 | The healthcare professional(s) and I discussed many things, but in relation to my way of managing the condition/situation/illness nothing has changed |  |  |  |  |  |  |  |  |  |  |  |  | The interaction seemed too superficial to have any significance for my situation/condition/illness | d31 |
| p18 | The healthcare professional(s) presented me with a wide range of options without anything concrete coming out of it |  |  |  |  |  |  |  |  |  |  |  |  |  | - |
| p19 | The healthcare professional(s) believed that I would acquire the skills to manage the challenges with my health on my own |  |  |  |  |  |  |  |  |  |  |  |  | The healthcare professional strengthened my confidence that I can handle challenges in my situation/condition/illness in the future | d27 |
| p20 | It was not just the condition that was in focus, but also my dreams and wishes about what I want more generally |  |  |  |  |  |  |  |  |  |  |  |  | There was focus on something that we both believed was important for me | d10 |
| p21 | The healthcare professional(s) decided without knowing about my difficulties living with the illness |  |  |  |  |  |  |  |  |  |  |  |  | *Both my experience of living with the situation/condition/illness and the healthcare professional’s professional knowledge influenced the decision about what to do | d15 |
| p22 | The healthcare professional(s) seemed to have given up on me |  |  |  |  |  |  |  |  |  |  |  |  | The healthcare professional seemed to have given up on me | d26 |
| p23 | Neither with the healthcare professional(s) nor myself were there any belief that we could improve my situation |  |  |  |  |  |  |  |  |  |  |  |  | *Through the interaction, the healthcare professional supported me in seeing my strong sides | d34 |
| p24 | The healthcare professional(s) underestimated how difficult it is to live with the illness |  |  |  |  |  |  |  |  |  |  |  |  | *The healthcare professional gave me room to explain how it really is for me to live with the situation/condition/illness | d11 |
| p25 | If the healthcare professional(s) suggested something that I didn’t want to do, I just remained silent |  |  |  |  |  |  |  |  |  |  |  |  |  | - |
| p26 | I avoided taking new medication because I was unhappy with the way it was prescribed* * If you haven’t been prescribed new medication, mark here __ |  |  |  |  |  |  |  |  |  |  |  |  |  | - |
| p27 | I used energy being in opposition to the healthcare professional(s) |  |  |  |  |  |  |  |  |  |  |  |  |  | - |
| p28 | I avoided going to appointments because I didn’t feel they were benefitting me |  |  |  |  |  |  |  |  |  |  |  |  | The interaction with the healthcare professional made me want to withdraw | d28 |
| p29 | I felt compelled to lie if I hadn’t done what we agreed |  |  |  |  |  |  |  |  |  |  |  |  | *I felt comfortable telling the healthcare professional if I, in my management of the situation/condition/illness, made different choices than agreed upon | d33 |
| p30 | If I hadn’t kept our agreements, I chose to cancel or not attend an appointment |  |  |  |  |  |  |  |  |  |  |  |  |  | - |
| p31 | The healthcare professional(s) seemed frustrated with me |  |  |  |  |  |  |  |  |  |  |  |  | The healthcare professional showed frustration towards me | d22 |
| p32 | I have managed by keeping life and disease apart |  |  |  |  |  |  |  |  |  |  |  |  | *I received adequate support to make my life and my situation/condition/illness fit together | d32 |
| p33 | I actually knew what the right thing to do was, but I didn't do it |  |  |  |  |  |  |  |  |  |  |  |  |  | - |
| p34 | I find it difficult to accept my condition/situation/illness |  |  |  |  |  |  |  |  |  |  |  |  |  | - |
| p35 | I got to explain in my own words what is difficult or challenging for me |  |  |  |  |  |  |  |  |  |  |  |  | There was room in the interaction for me to explain what was difficult or challenging for me | d4 |
| p36 | I got to work with some problems that I had been hiding away |  |  |  |  |  |  |  |  |  |  |  |  |  | - |
| p37 | During our conversations we quickly got to the heart of the matter |  |  |  |  |  |  |  |  |  |  |  |  |  | - |
| p38 | During the conversations we covered subjects that are usually difficult for me to talk about |  |  |  |  |  |  |  |  |  |  |  |  | During the interaction there was room to talk about difficult topics | d17 |
| p39 | Though I told the healthcare professional(s) about my difficulties, they didn’t try to clarify them |  |  |  |  |  |  |  |  |  |  |  |  | *The healthcare professional and I reflected together on my handling of the situation/condition/illness | d8 |
| p40 | The healthcare professional(s) and I circled around the problems without getting to the heart of the matter |  |  |  |  |  |  |  |  |  |  |  |  | *Through the interaction we managed to get to what I believe is the heart of the matter in my situation/condition/illness | d7 |
| p41 | I delivered information to the healthcare professional(s) without knowing what it would be used for in my situation |  |  |  |  |  |  |  |  |  |  |  |  | *I felt that the healthcare professional used knowledge about me in a constructive way | d35 |
| p42 | The healthcare professional(s) and I reached solutions by knowing one another’s different opinions |  |  |  |  |  |  |  |  |  |  |  |  |  | - |
| p43 | The healthcare professional(s) and I exchanged views on my situation in a meaningful way |  |  |  |  |  |  |  |  |  |  |  |  |  | - |
| p44 | The healthcare professional(s) gave me something to think about in relation to my way of managing my situation |  |  |  |  |  |  |  |  |  |  |  |  | The healthcare professional gave me something to think about in relation to my way of managing my situation/condition/illness | d24 |
| p45 | The dialogue with the healthcare professional(s) made me more aware of what can cause me problems |  |  |  |  |  |  |  |  |  |  |  |  | The interaction with the healthcare professional strengthened my understanding of my situation/condition/illness | d5 |
| p46 | I discovered that things were not going as well as I had thought  0 |  |  |  |  |  |  |  |  |  |  |  |  | Through the interaction I became aware of some things I can do myself to manage my situation/condition/illness | d18 |
| p47 | The conversations with the healthcare professional(s) made it easier for me to talk about things that are difficult with my family/friends/colleagues/other healthcare professionals |  |  |  |  |  |  |  |  |  |  |  |  |  | - |
| p48 | The healthcare professional(s) believed they could compare their own situation to mine |  |  |  |  |  |  |  |  |  |  |  |  | The healthcare professional mistakenly believed that s/he understood my situation/condition/illness based on their own experiences | d12 |
| p49 | The nature of the interaction was friendly |  |  |  |  |  |  |  |  |  |  |  |  | Irrelevant small talk took up too much time during the interaction | d14 |
| p50 | The healthcare professional(s) did not ask whether I was ready to follow their advice/prescriptions |  |  |  |  |  |  |  |  |  |  |  |  |  | - |
| p51 | I know the healthcare professional(s)’ view about my situation |  |  |  |  |  |  |  |  |  |  |  |  | Through the interaction I got a valuable insight into the healthcare professional’s thoughts about my way of managing the situation/condition/illness | d23 |
| p52 | The healthcare professional(s) asked what I think about my situation |  |  |  |  |  |  |  |  |  |  |  |  | The healthcare professional asked what I think about my situation/condition/illness | d6 |
| p53 | The care process gave me a clear picture of the benefits of managing the situation in an ideal way |  |  |  |  |  |  |  |  |  |  |  |  | The interaction gave me a clear picture of the benefits I can get from managing my situation/condition/illness in an ideal way in everyday life | d19 |
| p54 | During the care process it was the healthcare professional(s) who decided what should be discussed |  |  |  |  |  |  |  |  |  |  |  |  | During the interaction it was only the healthcare professional who decided what we should discuss | d20 |
| p55 | During the process it was clear that it was the health staff and not me that had responsibility for my situation |  |  |  |  |  |  |  |  |  |  |  |  | During the interaction, the healthcare professional appeared to take-over all responsibility for my situation/condition/illness | d25 |
|  | Items tested in each round | 46 | 41 | 39 | 36 | 35 | 32 | 33 | 33 | 36 | 36 | 36 | 36 |  |  |

**S2** Items evaluated by the expert panel and healthcare users in cognitive interviews

Note: Squares represent modifications to item content, circles represent item elimination, and an asterisk represents negative-to-positive modification to final draft wording. Final draft items (d1-36) were cross translated into English after round 8 of Danish cognitive interviews. For publication purposes only, all preliminary items were translated into English by a single translator.
Abbreviations: E, expert panel, T, translation
